# Effects of femoral bone defect morphology on initial polished tapered stem stability in massive defect model: a biomechanical study

**DOI:** 10.1186/s12891-019-2716-8

**Published:** 2019-08-01

**Authors:** Tohru Irie, Daisuke Takahashi, Tsuyoshi Asano, Tomohiro Shimizu, Ryuta Arai, Alaa Muhammad Terkawi, Yoichi M. Ito, Norimasa Iwasaki

**Affiliations:** 1 Department of Orthopaedic Surgery, Faculty of Medicine and Graduate School of Medicine, Hokkaido University, Kita 15, Nishi 7, Kita-ku, Sapporo, 060-8638 Japan; 2 Department of Biostatistics, Graduate School of Medicine, Hokkaido University, Sapporo, Japan

**Keywords:** Revision total hip replacement, Femoral bone defect, Circumferential metal mesh, Initial stem stability

## Abstract

**Background:**

Good outcomes have been reported in revision total hip replacement with massive segmental defects using impaction bone grafting with circumferential metal meshes. However, the morphology of defects that require a mesh is poorly defined. The purpose of this study was to evaluate the effects of a variety of segmental defects on load transmission to the proximal femur under both axial and rotational loads.

**Methods:**

Initial stability of the Exeter stem was investigated in a composite bone model using three medial bone defect morphologies: Long (length 5 cm × width 2 cm), Short (2.5 cm × 2 cm), Square (3.2 cm × 3.2 cm), Square with mesh (3.2 cm × 3.2 cm defect covered with metal mesh), and with no defect as control. Specimens (5 per group) were axially loaded and internally rotated up to 20° or to failure. Strain distributions of the femora were measured using a strain gauge.

**Results:**

All Square group specimens failed while rotation was increasing. In the other four groups, failure was not observed in any specimens. Mean torsional stiffness in the Long (4.4 ± 0.3 Nm/deg.) and Square groups (4.3 ± 0.3 Nm/deg.) was significantly smaller than in the Control group (4.8 ± 0.3 Nm/deg.). In the medio-cranial region, the magnitude of the maximum principal strain in the Square group (1176.4 ± 100.9) was significantly the largest (Control, 373.2 ± 129.5, *p* < 0.001; Long, 883.7 ± 153.3, *p* = 0.027; Short, 434.5 ± 196.8, p < 0.001; Square with mesh, 256.9 ± 100.8, p < 0.001). Torsional stiffness, and both maximum and minimum principal strains in the Short group showed no difference compared to the Control group in any region.

**Conclusions:**

Bone defect morphology greatly affected initial stem stability and load transmission. If defect morphology is not wide and the distal end is above the lower end of the lesser trochanter, it may be acceptable to fill the bone defect region with bone cement. However, this procedure is not acceptable for defects extending distally below the lower end of the lesser trochanter or defects 3 cm or more in width.

## Background

Revision of total hip replacement (THR) is challenging, though good clinical results have been reported [1, 2]. Progressive bone stock loss accompanies THR loosening. Moreover, removing the failed stem, and in some cases, cement, may result in further defects in the proximal femur. With polished tapered stems, proximal femoral bone defects have less effect on initial stem stability than with uncemented stems, so procedures equivalent to primary THR or the impaction bone grafting technique (IBG) without reinforcement are occasionally used in revision THR in cases of relatively small bone defects. While initial stem stability is essential in revision THR, a medial segmental defect in the femur can greatly attenuate initial stem stability [2–4]. Therefore, managing bone defects of the proximal femur is a central problem in revision THR.

Good clinical results have been reported for revision THR using IBG, which can also restore bone stock [1, 2]. However, major segmental defects, especially of the calcar femorale, have been reported to affect clinical outcomes [2]. When massive segmental defects are present, circumferential metal meshes are effective to create a stable reconstruction [3–5]. However, the specific extent of a bone defect that can cause severe reduction of initial stem stability remains unclear. Therefore, the morphology of defects that require circumferential metal mesh in revision THR is poorly defined.

For good long-term clinical results, not only initial stem stability but also load transmission to the femur is important [6, 7]. Uneven stress distribution leads to stress shielding. Furthermore, the fragility of periprosthetic bone caused by stress shielding increases the risk of mechanical loosening or periprosthetic fracture [7].

The hypothesis tested in this study was that proximal femur bone defect morphology would greatly influence initial stem stability and load transmission to the proximal femur. The purpose of this study was to evaluate the effects of a variety of segmental defects on load transmission to the proximal femur, under both axial and rotational loads, in order to analyze initial polished tapered stem stability.

## Methods

### Specimen preparations

**Fig. 1:**
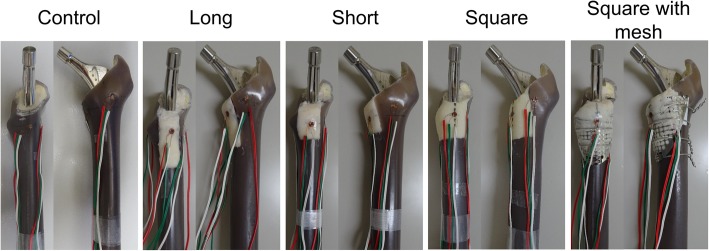
The composite bone and stem complex with three morphologies of proximal femur bone defect without circumferential metal mesh, square bone defect covered with metal mesh, and no-defect control

Initial stability of the Exeter stem (Exeter V40, 150 mm length, 44 mm offset, size no. 1, Stryker Orthopaedics, Mahwah, NJ) was investigated. A total of 25 composite Sawbones (medium left femur model 3403; Pacific Research Laboratories, Vashon, WA) were prepared. A standard femoral neck cut was made 1 cm proximal to the lesser trochanter. The bones were then broached using the appropriate Exeter broaching rasp. Following broaching, three kinds of proximal medial segmental defects were made (Fig. 1). In the Long group, the defect extended 5 cm distally from the resection level and had a width of 2 cm. This morphology was based on the method of Bolder et al. [3], and its length was equivalent to one-third of the stem length, creating a worst-case scenario in the distal direction. In the Short group, the defect morphology was defined as half of the length (2.5 cm) and the same width (2 cm) as the Long type defect. In the Square group, the defect morphology was a square with a side length of 3.2 cm, such that the area (theoretically 1024 mm^2^) was nearly equal to that of the Long type defect (theoretically 1000 mm^2^). This defect was created as a worst-case scenario in the anterior-posterior direction. Furthermore, the square type defect was reconstructed with a metal mesh (X-change Rim mesh-medium, Stryker Orthopaedics, Newbury, UK) as the Square with mesh group, which was fixed with 4 cerclage wires (diameter, 1.0 mm). After insertion of a cement plug, retrograde insertion of cement dough (CMW, DePuy Synthes, Warsaw, IN) was performed using a cement gun, followed by cement pressurization. The stem was then cemented, seated with the third marking at the level of the neck cut, using a stem centralizer. In groups with defects, the defects were covered by hand with gauze during the insertion of cement dough and stems to keep containment of the proximal part of the femur. We also evaluated stability in a no-defect control model. We tested 5 specimens in each group.

### Mechanical testing

The load-displacement behavior of the stem and the proximal femur strain distribution were measured using an Instron ElectroPuls E10000 (Instron Systems, Norwood, MA, dynamic linear load capacity: ±10 kN, dynamic torque capacity: ±100 Nm) mechanical testing machine. For strain gauge measurements, three rosettes (KFG-1-120-D17-11L3M2S, Kyowa Electronic Instruments Co., Ltd., Tokyo, Japan, strain measurement range limit at room temperature: 5.0%) were externally bonded to anterior, posterior, and medial surfaces 17 mm distally from the proximal edge of the femoral neck cut [5]. The wires from the rosettes were connected to a data logger (UCAM-550A, Kyowa Electronic Instruments Co., Ltd., Tokyo, Japan).

**Fig. 2:**
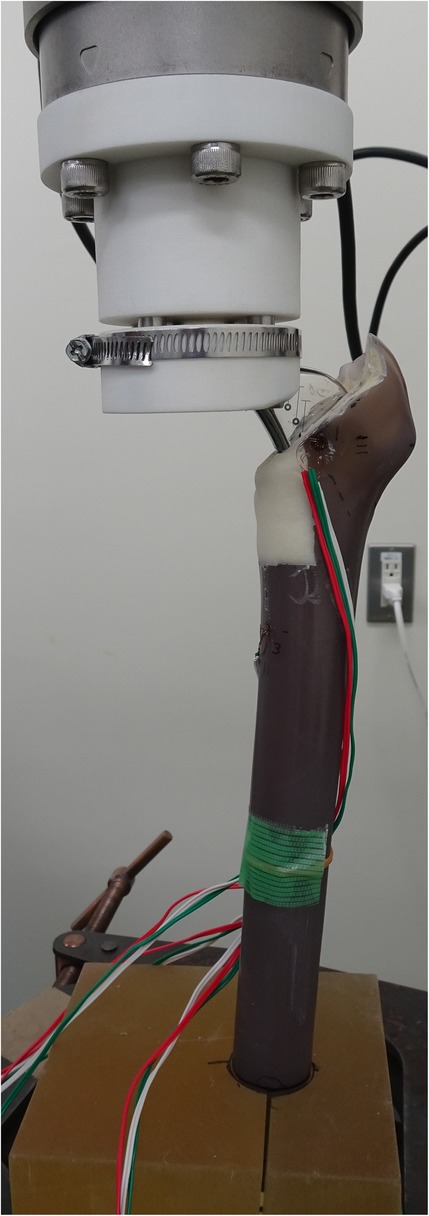
Experimental apparatus for the axial compression test and the rotational test combined with compression force. The proximal stem was clamped such that the center of rotation was located at the implant head

The composite bone and stem complex was positioned in the vertical loading axis of the machine at an angle of 15° to replicate the natural loading axis [5, 8]. The proximal stem was clamped such that the center of rotation was located at the implant head (Fig. 2). As preload, compression force of 1 kN was applied, and then the implant was internally rotated 5° while under compression, simulating the loading in a single leg stance. After two applications of this preload, the femora were loaded with compression force at a crosshead speed of 2 mm/min up to 3 kN, at which point the subsidence distance was measured. The femora were then tested in the rotational test while under compression. The compressive load of 3 kN was maintained, and the implant was internally rotated at a crosshead speed of 0.5°/sec up to 20° or the limit of complex failure. Complex failure was defined as fracture of the composite bone or fracture of the cement. The torque and the strain distribution of the proximal femur were measured during internal rotation. Fracture torque was defined as the maximum measured torque with complex failure. Fracture energy was calculated for the sample for which complex failure was obtained by numerically integrating the torque and angle measurements against time. Torsional stiffness was calculated from the torque-angle curve along the linear region from 5 Nm to 65 Nm of torque.

### Micro CT analysis

Following the experiment and after removing the stems, the proximal femur and cement complexes were evaluated with micro computed tomography (CT) (R mCT, Rigaku Corporation, Tokyo, Japan) at 20 μm/pixel to confirm the failure mechanism. The imaging conditions of micro CT were 80 kV and 100 μA.

### Statistical analysis

The delta was calculated using data from our pilot study. Because the delta was calculated to be 1.8, a sample size of 5 femurs in each group was required to provide 80% power. The subsidence distance during the axial compression test up to 3 kN, the torsional stiffness with torque from 5 to 65 Nm, the rotation angle at torque of 70 Nm, and the maximum and minimum principal strain of the antero-cranial, postero-cranial, and medio-cranial regions of the femur at torque of 70 Nm were each statistically compared. All statistical analyses were performed using JMP Pro 14 software (SAS Institute Japan, Tokyo, Japan). Pairwise comparisons among each of the 5 groups were performed using ANOVA with Tukey’s post hoc test. Data is presented as mean ± SD and the corresponding 95% confidence intervals. A *p* value < 0.05 was considered significant.

## Results

### Axial compression test

In all groups, complex failure was not observed in any specimens. Mean subsidence distance under the axial compression test was not significantly different among the five groups (Table 1).

**Table 1:** Subsidence distance during the axial compression test up to 3 kN, torsional stiffness with torque from 5 to 65 Nm under the rotational test, and internally rotated angle at torque of 70 Nm with 3 kN of compressive load

Defect morphology (length × width) (cm) (area) (mm^2^)	Control (none) (0)	Long (5 × 2) (1000)	Short (2.5 × 2) (500)	Square (3.2 × 3.2) (1024)	Square with Mesh (3.2 × 3.2) (1024)	*P* value
Subsidence distance (mm)	2.6 ± 0.1 (2.4 to 2.8)	2.5 ± 0.1 (2.4 to 2.6)	2.5 ± 0.1 (2.4 to 2.7)	2.6 ± 0.3 (2.3 to 2.9)	2.5 ± 0.3 (2.2 to 2.9)	0.97
Torsional stiffness (Nm/deg.)	4.8 ± 0.3 (4.5 to 5.2)	4.4 ± 0.3* (4.0 to 4.7)	4.5 ± 0.2 (4.3 to 4.8)	4.3 ± 0.3* (4.0 to 4.7)	4.6 ± 0.2 (4.4 to 4.8)	0.03
Internally rotated angle (deg.)	15.0 ± 1.2 (13.5 to 16.4)	15.4 ± 0.6 (14.6 to 16.2)	15.7 ± 0.4 (15.2 to 16.2)	15.7 ± 0.5 (15.2 to 16.3)	15.1 ± 0.4 (14.7 to 15.6)	0.29

Values are expressed as mean ± SD with 95% confidence interval in parentheses; * significant compared with the Control group.

### Rotational test

**Fig. 3:**
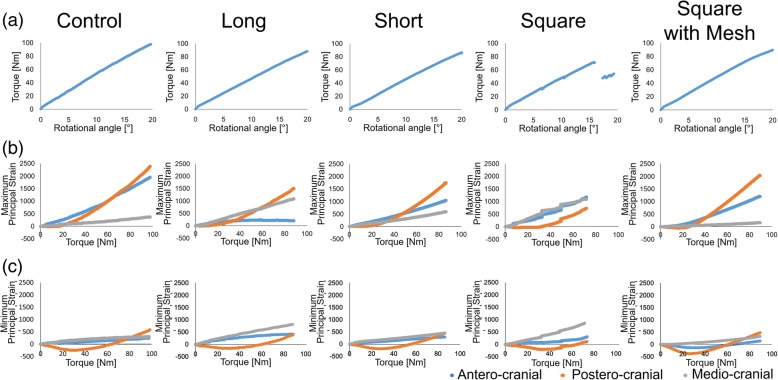
(**a**) Representative rotational angle–torque curves under the rotational test combined with compression force in each group. (**b**) Representative torque–maximum principal strain curves under the rotational test combined with compression force in each group. (**c**) Representative torque–minimum principal strain curves under the rotational test combined with compression force in each group. Both the rotational angle-torque curve and the torque-strain curves in the Square group showed two steps before complex failure

Mean torsional stiffness with torque from 5 to 65 Nm in both the Long group (4.4 ± 0.3 [95% confidence interval {CI}, 4.0 to 4.7] Nm/deg.) and the Square group (4.3 ± 0.3 [95% CI, 4.0 to 4.7] Nm/deg.) was significantly smaller than in the Control group (4.8 ± 0.3 [95% CI, 4.5 to 5.2] Nm/deg.), although the stiffness did not differ among the Control group, the Short group (4.5 ± 0.2 [95% CI, 4.3 to 4.8] Nm/deg.) and the Square with mesh group (4.6 ± 0.2 [95% CI, 4.4 to 4.8] Nm/deg.) (Table 1, Fig. 3a). Mean internal rotation angles at torque of 70 Nm were not significantly different among the five groups (Table 1, Fig. 3a).

**Fig. 4:**
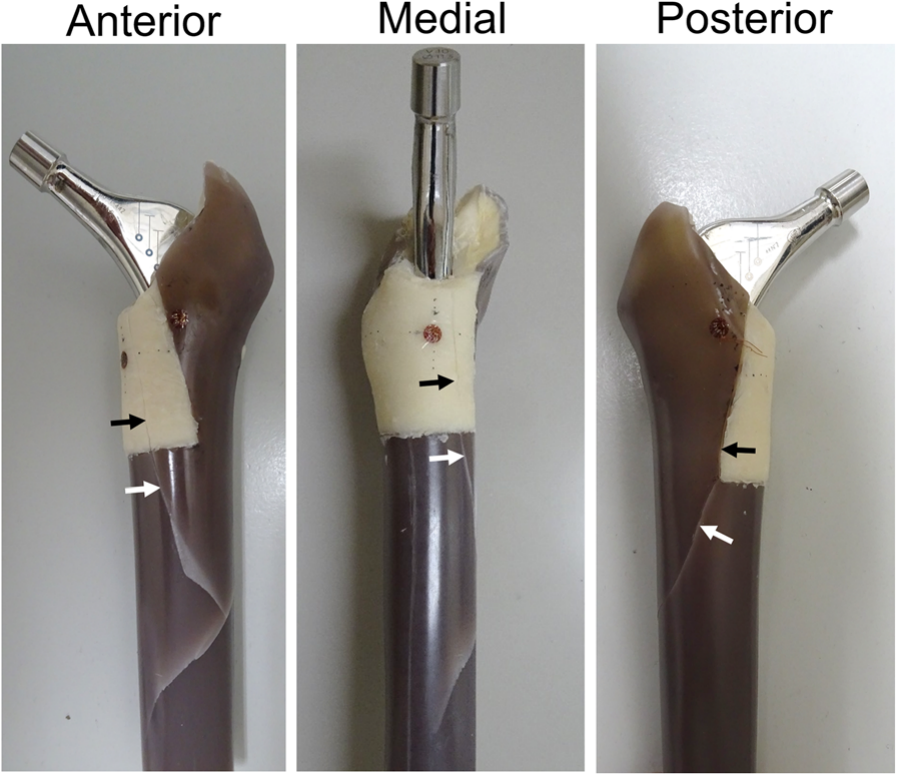
Representative macro findings of a Square group specimen after mechanical testing. The black arrows denote the fracture lines in the bone cement. The white arrows denote the fracture lines in the composite bone, which are continuous with fracture lines in the bone cement

In the Square group, all specimens failed at the proximal part of the femur while rotation was increasing up to 20°. Mean fracture torque was 74.0 ± 3.7 (95% CI, 69.4 to 78.6) Nm and mean fracture energy was 13.6 ± 2.0 (95% CI, 11.1 to 16.0) J (Table 2). All fractures showed a spiral fracture pattern. In all Square group specimens, fracture lines were observed in the distal direction continuing from the medio-cranial region of the bone cement into the composite bone (Fig. 4). In the other four groups, complex failure was not observed in any specimens.

**Table 2:** Number of steps observed in the rotation angle-torque curve by complex failure, torque when the first step is observed, fracture torque, and fracture energy in Square group

	Mean ± SD (95% CI)
Number of steps (specimens)	2 steps: 4, 3 steps: 1
Torque of the first step (Nm)	37.6 ± 5.7 (30.5 to 44.6)
Fracture torque (Nm)	74.0 ± 3.7 (69.4 to 78.6)
Fracture energy (J)	13.6 ± 2.0 (11.1 to 16.0)

Values are expressed as mean ± SD with 95% confidence interval in parentheses.

In all Square group examinations, the rotational angle-torque curve showed two or three steps before complex failure (Table 2, Fig. 3a). The mean torque of the first step was 37.6 ± 5.7 (95% CI, 30.5 to 44.6) Nm. In the Square group, the torque-maximum principal strain curve and torque–minimum principal strain curve under the rotational test also showed two or three steps that correspond to the steps on the rotational angle-torque curve (Fig. 3b and c).

**Fig. 5:**
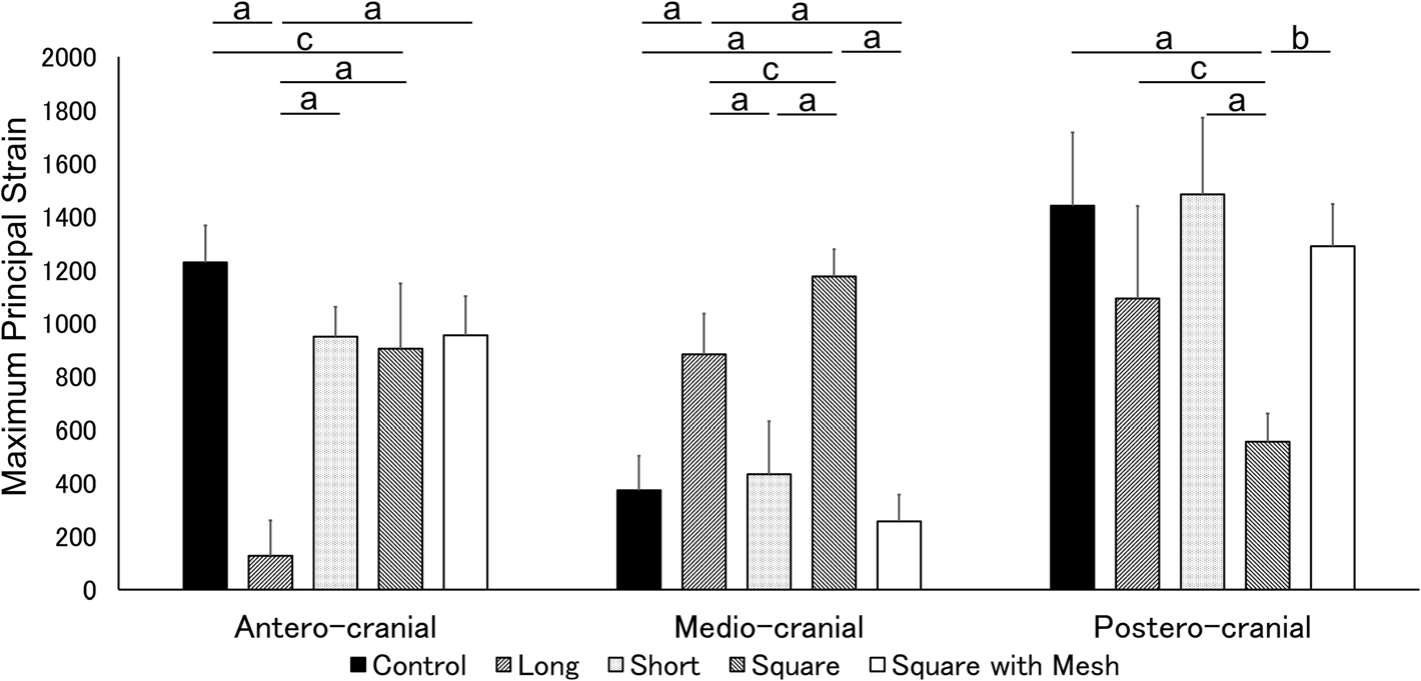
Magnitude of maximum principal strain at 70 Nm of torque with 3 kN of compressive load. a: *p* < 0.001, b: *p* < 0.01, c: *p* < 0.05

**Fig. 6:**
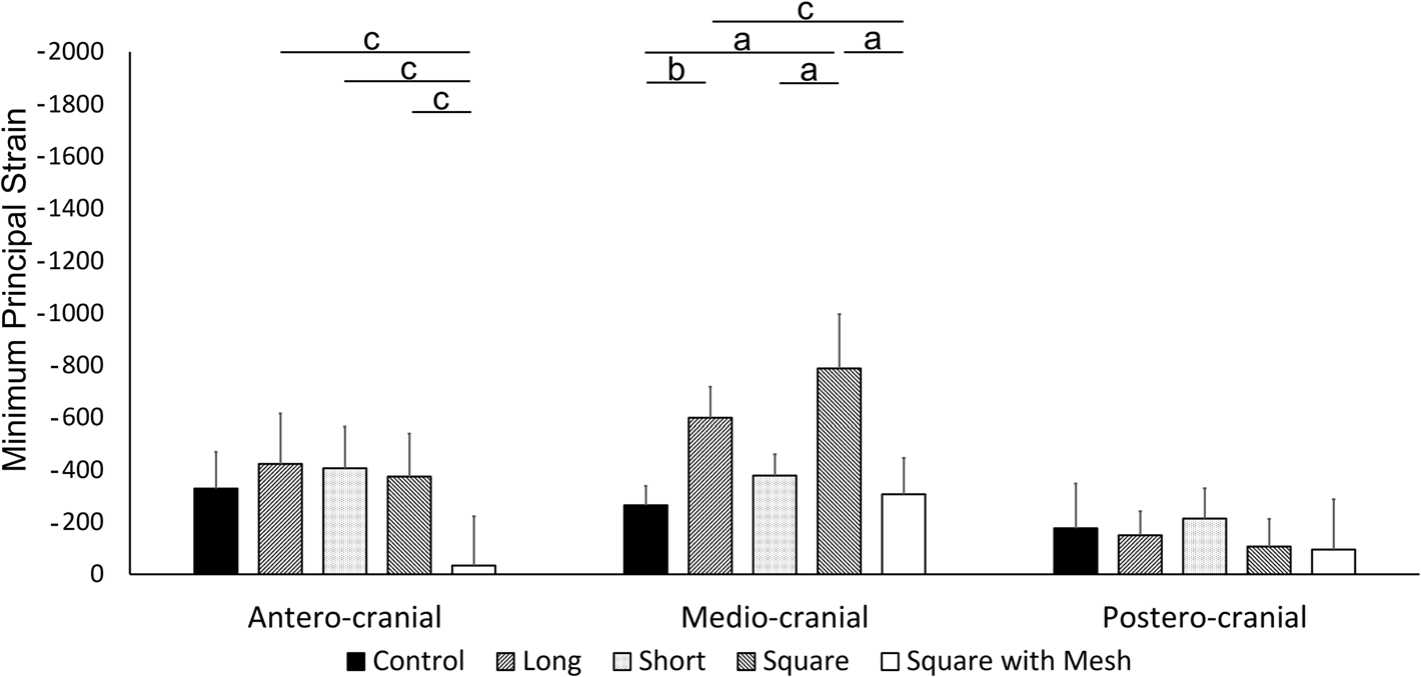
Magnitude of minimum principal strain at 70 Nm of torque with 3 kN of compressive load. a: *p* < 0.001, b: p < 0.01, c: p < 0.05

In the antero-cranial region, the magnitude of the maximum principal strain at torque of 70 Nm in the Long group (127.2 ± 133.0 [95% CI, − 37.9 to 292.3]) was significantly the smallest among the five groups (Control group, 1229.6 ± 137.1 [95% CI, 1059.4 to 1399.8], *p* < 0.001; Short group, 950.6 ± 111.2 [95% CI, 812.4 to 1088.7], p < 0.001; Square group, 904.9 ± 244.8 [95% CI, 600.9 to 1208.9], p < 0.001; Square with mesh group, 955.1 ± 147.6 [95% CI, 771.9 to 1138.4], p < 0.001). This magnitude in the Square group was significantly smaller than the Control group (*p* = 0.034). However, there were no significant differences among the Short group, the Square group, and the Square with mesh group (Fig. 5). In the same region, the absolute value of the minimum principal strain in the Square with mesh group (− 32.8 ± 190.3 [95% CI, − 269.0 to 203.4]) was significantly smaller than in the Long group (− 423.0 ± 192.0 [95% CI, − 661.3 to − 184.6], *p* = 0.013), Short group (− 406.9 ± 158.1 [95% CI, − 603.2 to − 210.6], *p* = 0.018), and Square group (− 374.6 ± 165.5 [95% CI, − 580.0 to − 169.1], *p* = 0.035). However, there were no significant differences among the Control (− 328.3 ± 140.9 [95% CI, − 503.2 to − 153.4]), Long, Short, and Square groups (Fig. 6).

In the medio-cranial region, the magnitude of the maximum principal strain in the Square group (1176.4 ± 100.9 [95% CI, 1051.2 to 1301.7]) was significantly the largest among the five groups (Control group, 373.2 ± 129.5 [95% CI, 212.4 to 533.9], *p* < 0.001; Long group, 883.7 ± 153.3, [95% CI, 693.3 to 1074.1], *p* = 0.027; Short group, 434.5 ± 196.8, [95% CI, 190.2 to 678.9], p < 0.001; Square with mesh group, 256.9 ± 100.8, [95% CI, 131.8 to 382.0], p < 0.001) (Fig. 5) and the absolute value of the minimum principal strain in the Square group (− 788.8 ± 208.1 [95% CI, − 1047.2 to − 530.3]) was significantly larger than in the Control group (− 264.4 ± 75.0 [95% CI, − 357.5 to − 171.3], *p* < 0.001), Short group (− 379.2 ± 80.9, [95% CI, − 479.6 to − 278.7], p < 0.001), and Square with mesh group (− 307.4 ± 139.0, [95% CI, − 480.0 to − 134.8], p < 0.001) (Fig. 6). In the same region, the magnitude of the maximum principal strain in the Long group was significantly larger than in the Control group (p < 0.001), the Short group (*p* = 0.001), and the Square with mesh group (p < 0.001) (Fig. 5). Additionally, the absolute value of the minimum principal strain in the Long group (− 598.4 ± 118.7 [95% CI, − 745.9 to − 451.0]) was significantly larger than in the Control group (*p* = 0.006) and the Square with mesh group (*p* = 0.019) (Fig. 6).

In the postero-cranial region, the magnitude of the maximum principal strain in the Square group (555.7 ± 105.1 [95% CI, 425.2 to 686.1]) was significantly the smallest (Control group, 1440.3 ± 275.5 [95% CI, 1098.2 to 1782.3], p < 0.001; Long group, 1093.1 ± 347.0, [95% CI, 662.3 to 1524.0], *p* = 0.022; Short group, 1483.5 ± 288.4, [95% CI, 1125.4 to 1841.6], p < 0.001; Square with mesh group, 1288.7 ± 158.1, [95% CI, 1092.4 to 1485.0], *p* = 0.002) (Fig. 5). In the same region, the absolute values of the minimum principal strains were not significantly different among the five groups (Control group, − 176.7 ± 171.5 [95% CI, − 389.6 to 36.2]; Long group, − 150.2 ± 92.0 [95% CI, − 264.4 to − 36.0]; Short group, − 213.6 ± 115.8 [95% CI, − 357.4 to − 69.8]; Square group, − 106.5 ± 105.5 [95% CI, − 237.6 to 24.5]; Square with mesh group, − 122.2 ± 192.7 [95% CI, − 361.5 to 117.1]) (Fig. 6).

### Micro CT evaluation

**Fig. 7:**
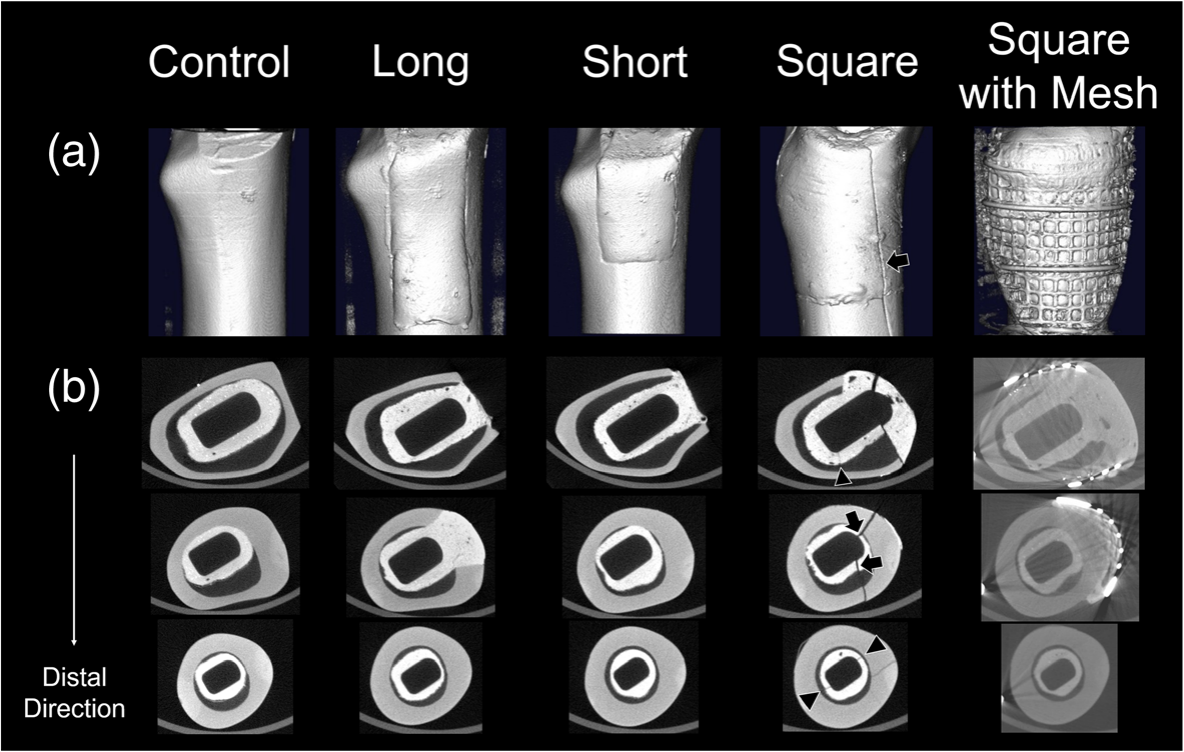
Representative micro CT imaging of the proximal region of the composite bone after removing stem. (a) Three-dimensional imaging of the cranial-medial region. (b) Axial imaging of the proximal region of the composite bone and bone cement. The arrowheads denote cracks in the cement mantle that were different from the composite bone fracture lines. The arrows denote the cement fracture lines, which are continuous with fracture lines in the composite bone

Micro CT showed some cement cracks different from the composite bone fracture lines in all Square group specimens (Fig. 7). In the other four groups, neither fracture lines nor cement cracks were observed in any specimens.

## Discussion

The purpose of this study was to evaluate the effects of a variety of segmental defects on load transmission to the proximal femur, in order to analyze initial polished tapered stem stability. Bolder et al. reported that the presence of a large medial segmental defect in the femur specifically reduces prosthetic stability, and that metal mesh was effective in creating a stable stem construction in a goat femur model [3]. However, the specific extent of a bone defect that can cause severe reduction of initial stem stability remains unclear. Therefore, it was unknown what type of defect requires reinforcement, such as metal meshes. In the present study, all specimens in the Square group failed while rotation was increasing up to 20°, and fracture lines were observed in the distal direction continuing from the medio-cranial region of the bone cement into the composite bone. Additionally, both the rotational angle-torque curve and the torque-strain curve showed two or three steps before complex failure, and the absolute values of both the maximum and minimum principal strains in the medio-cranial region were significantly the largest in the Square group, except that the absolute value of the minimum principal strain showed no difference compared to the Long group. In addition, the mean torsional stiffness with torque from 5 to 65 Nm in the Square group was significantly smaller than in the Control group. Furthermore, micro CT showed some cracks in the cement mantle that were different from the composite bone fracture lines.

The elastic modulus and ultimate strength of polymethyl methacrylate are one-third to one-seventh that of cortical bone [9, 10]. Considering this, it is reasonable to suppose that the cement cracks occurred during rotation before breakage of the composite bone. Because the first step was observed at a mean torque of 37.6 ± 5.7 Nm, there is a possibility that cement cracking may occur even with weak torque in cases of wide defects such as the Square group defect, although mean fracture torque was 74.0 ± 3.7 Nm. Kaneuji et al. reported that the greatest compressive force at the bone-cement interface was observed at the proximal medial region in an in vitro polished tapered stem model [11, 12]. The present study suggests that, if revision THR is performed without reinforcement despite a morphologically wide bone defect, the mechanical stress is concentrated excessively in the bone cement, and this excessive mechanical stress may cause early periprosthetic fracturing, cement cracking, and/or lead to early mechanical loosening. These results suggest that, even if a bone defect fits Paprosky classification type 2A [13, 14], when wide medial segmental defects more than 3 cm in width are present, reinforcement is necessary to prevent periprosthetic fracture or early mechanical loosening.

Although the areas of the defects of the Long group and the Square group were nearly equal, neither complex failure nor cement cracking was observed in the Long group. The present study showed that distally extended bone defects categorized as Paprosky classification type 2C, such as the Long group defect, affect initial stem stability less than wider defects, such as the Square group defect.

In the medio-cranial region, the magnitude of the maximum principal strain in the Long group was significantly larger than in the Control group, the Short group, and the Square with mesh group. In the same region, the absolute value of the minimum principal strain in the Long group was also significantly larger than in the Control group, and the Square with mesh group. Meanwhile, in the antero-cranial region, the magnitude of the maximum principal strain in the Long group was significantly the smallest among the five groups, although the absolute value of the minimum principal strain showed no difference except for the Square with mesh group.

It has been recognized that polished tapered stems slip in the cement and create considerable radial compressive loads at the bone-cement interface [11, 12, 15]. In the case of a distally extended defect, the tensile stress in the anterior region due to internal rotation may be absorbed at the bone defect region and not transmitted to the anterior part of the femur. Such imbalance of the stress distribution may cause stress shielding of the anterior proximal bone, resulting in bone fragility [16, 17]. The quality of periprosthetic bone determines the risk of periprosthetic fracture [7], and a high incidence of early periprosthetic fractures is associated with polished tapered stems [8, 18–20]. Furthermore, our results showed that the mean torsional stiffness in the Long group was significantly smaller than in the Control group. If revision THR with defects that extend below the lower end of the lesser trochanter is performed without reinforcement, this bone defect may cause loosening or periprosthetic fracture postoperatively. The present results suggest that it is preferable to perform IBG with circumferential metal mesh for defects with such morphology to obtain good long-term clinical results.

The torsional stiffness in the Short group did not differ from that of the Control group. Additionally, in the medio-cranial region, the magnitude of the maximum principal strain in the Short group was significantly smaller than in the Long and the Square groups and the absolute value of the minimum principal strain was also significantly smaller than in the Square group. Furthermore, the absolute values of both the maximum and minimum principal strains in the Short group showed no difference compared to the Control group in any region. These results suggest that, if defect morphology is not wide and the distal end is at or above the lower end of the lesser trochanter, attenuation of initial stem stability is minimal. This is consistent with reports that the extent of the bone defect affects the outcome of revision THR [2].

The procedure of femoral IBG is recognized as technically demanding, and one of the reasons for this is the frequent occurrence of intraoperative periprosthetic femoral fractures [1, 21, 22]. Additionally, a higher incidence of postoperative femoral fractures, excessive femoral component subsidence, dislocation, and deep joint infections has been reported in femoral reconstruction with IBG compared to primary THR [21, 23, 24]. Hence, if bone defect morphology is limited, similar to the Short group defect, a surgical procedure to fill the bone defect region with bone cement without IBG can be considered, especially for patients with high risk of surgical site infection, or patients who cannot tolerate large surgical invasion. Although evaluation of bone defects before surgery and preoperative planning are very important for revision THR [23], it is widely recognized that preoperative evaluation of bone defects is difficult [25, 26], and that new bone defects may occur at the time of stem or bone cement removal [27, 28]. An IBG procedure is ideal in all cases of bone defect. However, circumstances such as the absence of an experienced surgeon, or a lack of graft bone or materials required for IBG may occur. In such cases, it may be acceptable to fill the bone defect region with bone cement, if it is limited to a morphology comparable to our Short group defect. However, this procedure is not acceptable for cases with larger defects, as demonstrated by our Long and Square groups.

It is difficult to accurately reproduce actual clinical conditions with an experimental study. High torsional loads occur due to forces on the femoral head during stair climbing or rising from a chair, which can cause critical micromotion and may result in stem loosening [4, 20, 29]. Thus, it is important to evaluate initial stem stability not only against axial load, but also against torsional load [30, 31]. To reproduce actual clinical conditions as closely as possible, the axial load was applied and then maintained, and a rotational test with the femoral head center at the center of rotation was performed. This is a strength of the present study.

There were several limitations. First, a composite Sawbone model was used in this study. Malkani et al. pointed out that torsional strength and stiffness were primarily dependent on variability in bone quality in individual specimens [32]. Since individual differences among samples cannot be avoided in cadaveric bones and animal bones, a composite bone model was used to minimize intersample variability. Although this Sawbone is a composite bone for mechanical testing, it does not have cancellous bone, which may affect cementing and initial stability. Second, IBG was not performed in the Square with mesh group. Since the femoral IBG procedure is technically demanding, there is a risk of increasing intersample variability. Additionally, initial stem stability in IBG has already been sufficiently evaluated [3–5]. Since cortical bone has much stronger mechanical properties than cancellous bone [33] or impacted bone [34, 35], proximal femoral cortical bone defects in particular affect initial stability. Although we recognized that tight packing of the bone graft may affect initial stability, the primary objective of this study was to clarify the effects of a variety of segmental defects on stem stability. Therefore, the focus was on evaluating the effect of segmental defect morphology. Third, mechanical stress distribution on composite bone was not directly measured. The direct measurement of bone stress distribution, especially around cemented orthopedic implants, remains challenging. Kaneuji et al. reported a method for evaluating compressive force at the bone-cement interface, which has been recognized as excellent [11, 12]. However, this method requires complete fixation of the bone in the device, embedding the entire femur except for the proximal part. Hence, this set up could prevent femoral bone failure, which was the primary outcome variable for the present study. Additionally, drilling holes into the femur to insert a measuring rod would alter its structural integrity, thereby influencing the outcome variables of complex failure and bone strain. Considering that complex failure or internal rotation angle 20° were the endpoints of our rotation test, it is reasonable to assume that load transmission to the femur was evaluated by measuring the composite bone strain using a strain gauge.

In the present study, complex failure was evaluated only in the Square group. In our preliminary experiments, the torque at a rotational angle of 20° in the no-defect control model was about 100 Nm. The torque capacity of the mechanical testing machine used in this study was ±100 Nm. Hence, the endpoint of the rotation test was set to complex failure or an internal rotation angle of 20°. Therefore, we did not analyze fracture torque and fracture energy in the Control, Long, Short, and Square with mesh groups. This was a major limitation in our study. Fracture torque and fracture energy are important indicators to evaluate the effects of the segmental defects on load transmission and initial stem stability. However, they can be greatly influenced by stem design and size, stem insertion depth, and the conditions of the mechanical test, among other things [36]. Therefore, comparing the fracture torque and fracture energy of the Square group with other research results found under different experimental conditions may mislead the interpretation of the results. Thus, further investigations under the same conditions are necessary to evaluate the effects of long type and short type defects on fracture torque and fracture energy.

## Conclusions

The present study showed that the morphology of proximal femur bone defects greatly affected initial polished tapered stem stability and load transmission to the proximal femur. Even with proximal medial segmental bone defects of approximately the same size, wider defects in the anterior and posterior attenuate initial stability significantly more than vertically long defects. In contrast, if defect morphology is not wide and the distal end is at or above the lower end of the lesser trochanter, attenuation of load transmission and initial stem stability are minimal. In such cases, it may be acceptable to fill the bone defect region with bone cement. However, this procedure is not acceptable for cases with bone defects extending distally below the lower end of the lesser trochanter or wide defects with a width of 3 cm or more.

## Data Availability

The datasets used in the present study are available from the corresponding author on reasonable request.

## Abbreviations

CT: Computed tomography
IBG: Impaction bone grafting
THR: Total hip replacement
